# Sensitization of ovarian carcinoma cells to Bcl-x_L_-targeting strategies through indirect modulation of Mcl-1 activity by MR22388, a molecule of the tripentone family

**DOI:** 10.1186/1757-2215-6-38

**Published:** 2013-06-05

**Authors:** Julie Tomasina, Aurélie Malzert-Freon, Florence Giffard, Emilie Brotin, Marie-Hélène Louis, Edwige Abeilard, Sylvain Rault, Pascal Gauduchon, Laurent Poulain

**Affiliations:** 1 Normandie University, Caen, France; 2 UNICAEN, BioTICLA EA 4656, F 14032, Caen, France; 3 Centre de Lutte Contre le Cancer F. Baclesse, F-14032, Caen, France; 4 UNICAEN, CERMN EA 4258, F-14032, Caen, France

**Keywords:** Tripentone MR22388, Ovarian cancer, Apoptosis, Chemoresistance

## Abstract

**Background:**

Our work has been carried out in the context of the therapeutic failure in ovarian carcinoma, which remains the leading cause of death by gynecologic malignancy. In these tumours, recurrence and subsequent acquired chemoresistance constitute major hurdles to successful therapy. Here we studied the interest of a member of the tripentone chemical family, MR22388, for the treatment of chemoresistant ovarian cancer cells.

**Findings:**

MR22388 activity has been assessed *in vitro* on cisplatin-resistant (SKOV3 and IGROV1-R10) ovarian cancer cell lines by conventional analysis, alone or combined to a BH3-mimetic molecule, ABT-737. MR22388 exerts its activity on cisplatin resistant cells, and we showed that it induces a decrease of the Mcl-1 anti-apoptotic protein expression. Considering our previous work demonstrating that the efficiency of Bcl-x_L_ targeting strategies is conditioned to the concomitant inhibition of Mcl-1 we studied the interest of the association of this MR22388 with ABT-737, and showed that this combination was highly cytotoxic in chemoresistant cells.

**Conclusions:**

This work thus opens new perspectives for the use of this promising molecule for the treatment of highly chemoresistant ovarian cancer cells and for sensitization of emerging Bcl-x_L_ targeting strategies such as the use of BH3-mimetic molecules.

## Findings

### Background

Ovarian cancer is the leading cause of death by gynecological malignancies [1]. Standard first line treatment (debulking surgery followed by platinum/taxane based chemotherapy) and various second line chemotherapies appear generally unable to cure the disease [2], the therapeutic failure being mainly due to a multifactorial intrinsic or acquired chemoresistance [3,4]. Thus, there is an urgent need of new strategies for the treatment of refractory disease.

Our previous work showed that platinum resistance was associated with the capacity of ovarian cancer cells to maintain a high level of two anti-apoptotic proteins of the Bcl-2 family, Bcl-x_L_ and Mcl-1 [5]. These proteins cooperate to protect ovarian cancer cells against apoptotic cell death and their concomitant inhibition leads to massive cell death even in the absence of chemotherapy. We also demonstrated that Mcl-1 down-regulation or inactivation leads to a strong sensitization to Bcl-x_L_-targeting strategies, such as treatment by the BH3-mimetic ABT-737 [6-8].

The 8H-thieno[2,3-*b*]pyrrolizinones, also named tripentones, constitute a promising family of novel compounds for cancer therapeutics [9]. Among them, the 3-(3-hydroxy-4-methoxyphenyl)-8H-thieno[2,3-*b*]pyrrolizin-8-one (MR22388, abbreviated TP) has shown a significant cell growth inhibitory activity in the nanomolar range over a panel of human tumor cell lines, including ovarian carcinoma cells, and can be considered as the *lead* of this family [9,10]. To date, the precise mechanisms of action of MR22388 remain unknown.

Here we investigated the cytotoxic activity of MR22388 on cisplatin-resistant ovarian carcinoma cells and its potential as a modulator of the expression or activity of Bcl-x_L_ and Mcl-1. We also tested its cytotoxic activity when used in combination with ABT-737.

## Materials & methods

### Chemicals

MR22388 [11] was purchased from Syntheval (Caen, France). ABT-737 was purchased from Selleckchem (Euromedex, Souffelweyersheim, France).

### Cell culture and treatment

SKOV3 cell line obtained from ECACC (Cerdic, Nice, France) and chemoresistant IGROV1-R10 subline obtained from IGROV1 parental cells [12] were cultured as described [13]. Cells were continuously treated with MR22388 or ABT-737. Cell viability was determined by Trypan blue exclusion assay using Cedex XS device (Roche, France).

### Real-time cellular activity assay (xCELLigence)

Compound-mediated cytotoxicity was monitored using Real-Time Cell Analyzer (RTCA) multi-plate Instrument, xCELLigence System (Roche Applied Science, Mannheim, Germany) [13]. The cell index reflects changes in cell viability as described elsewhere [14]. Briefly, cells were left to grow for 24 h before treatment in 96-well E-Plate and impedance was continuously measured until the end of the treatment. Standard deviations of well replicates were analyzed with the RTCA Software.

***RNA isolation and quantitative reverse transcripase-PCR (RT-PCR)*** were performed as described elsewhere [7].

***Determination of cellular DNA content*** was performed by flow cytometry as described elsewhere [13].

***Preparation of cell extracts and western blot analysis*** were performed as described elsewhere [13]. The following antibodies were used: PARP, Caspase-3 and Bcl-x_L_ (Cell Signaling Technology by Ozyme, Saint-Quentin-en-Yvelines, France), MCL-1 (Santa Cruz by CliniSciences, Nanterre, France), phospho(Ser62)-Bcl-x_L_ (AssaybioTech by Tebu-bio, Le Perray-en-Yvelines, France) and Actin (Sigma-Aldrich, Saint-Quentin Fallavier, France).

## Results

### Effect of MR22388 on the proliferation and apoptosis of SKOV3 and IGROV1-R10 cisplatin-resistant cell lines

**Figure 1:**
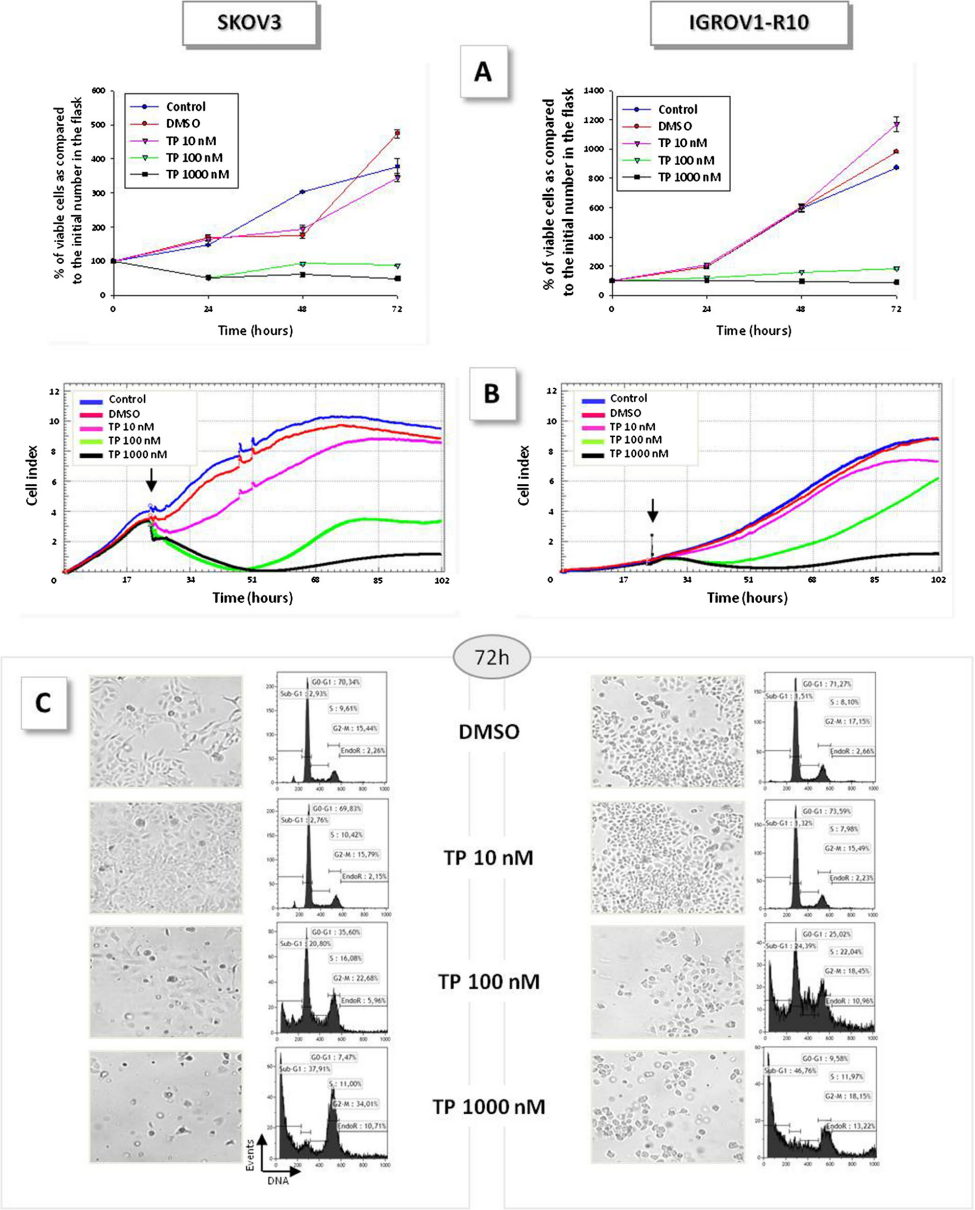
**MR22388 exerts a cytostatic and cytotoxic effect on cisplatin-resistant cell lines.** Exponentially growing SKOV3 and IGROV1-R10 cells were treated with MR22388 at 10, 100 and 1000 nM. [**A**] Percentages of cell viability, assessed by trypan blue exclusion test after 24, 48 and 72 h exposure to MR22388, as compared to the initial number of viable cells in the flask. [**B**] Real time analysis of cellular activity of MR22388 obtained using the xCELLigence system. Cells were grown for 24 h, then treated with MR22388 (10, 100 and 1000 nM). Cell index was recorded every 2 h. The results are the mean of three replicates. [**C**] DNA content histograms and cellular morphologies, assessed 72 h after the beginning of the MR22388 exposure.

MR22388 induced a strong cytostatic effect from 100 nM on both cell lines, with a slight dose-effect between 100 and 1000 nM (Figures 1A and 1B). Moreover, MR22388 exposure induced an S phase elongation in IGROV1-R10 cells and a G2-M phase blockade in both cell lines (Figure 1C). Massive cell death occurred in a dose-dependent manner after a 72 h exposure to MR22388 (Figure 1C), as shown by cell detachment and sub-G1 peak appearance on DNA content histograms. PARP and Caspases 9 and 3 cleavages have been observed in a time and concentration dependent manner (data not shown). However, the exposure to 100nM MR22388 allowed a slow recovery of a proliferating cell population in both cell lines (Figure 1A). At this concentration, MR22388 also induced a transient cell detachment and rounding of SKOV3 cells that was not associated to massive cell death, responsible for a transient decrease cell index on impedancemetry curves (Figure 1B, left panel). After 24 h, the cells recovered a normal adhesion but were unable to proliferate, the cell index thus reaching a plateau after 48 h.

#### MR22388 modulates MCL-1 expression and Bcl-x_L_ phosphorylation

**Figure 2:**
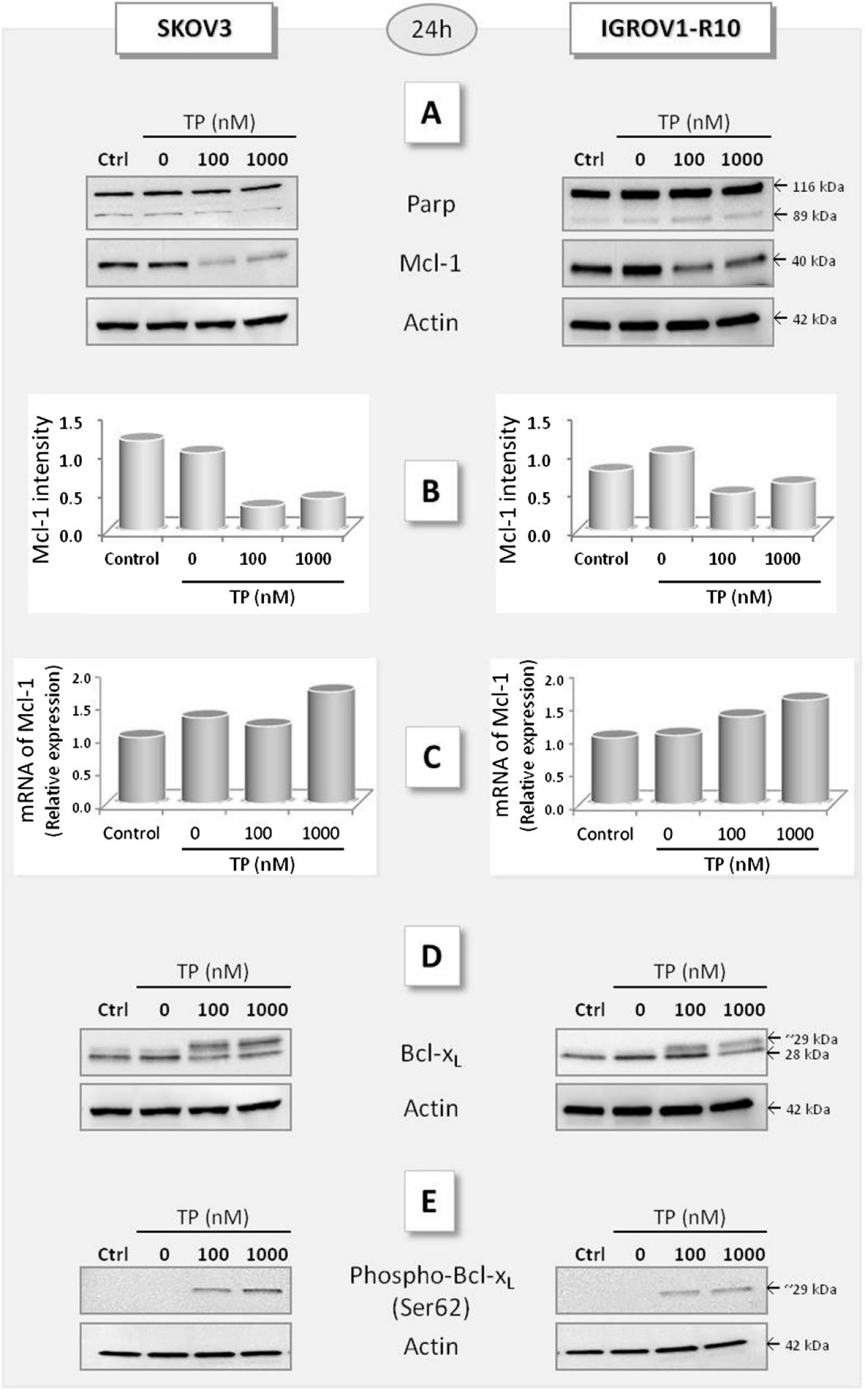
**MR22388 modulates MCL-1 expression and Bcl-x**_**L **_**phosphorylation.** Cells were treated with MR22388 (100 and 1000 nM) during 24 h. [**A**] PARP cleavage and Mcl-1 expression, analyzed by western blot. Actin was used as loading control. [**B**] Mcl-1 expression quantified by Image J software. The relative intensity of each lane was calculated as compared to the DMSO sample. [**C**] Mcl-1 mRNA was quantified by qRT-PCR. [**D**] Bcl-x_L_ expression and [**E**] Ser62-phospho-Bcl-x_L_ detected by western blot.

A 24 h exposure to MR22388 led to a half-decrease of Mcl-1 expression in both cell lines from 100 nM (Figure 2A and 2B), in absence of cell death. This Mcl-1 protein disappearance was not associated to transcriptionnal down-regulation (Figure 2C). Otherwise, at the same time, MR22388 did not modified Bcl-x_L_ expression but induced the appearance of a slower-migrating Bcl-x_L_ band evocative of a phosphorylated form (Figure 2D). We showed that this band was at least in part constituted by a Ser62-phosphorylated form of Bcl-x_L_ (Figure 2E).

#### MR22388 sensitizes to ABT-737

**Figure 3:**
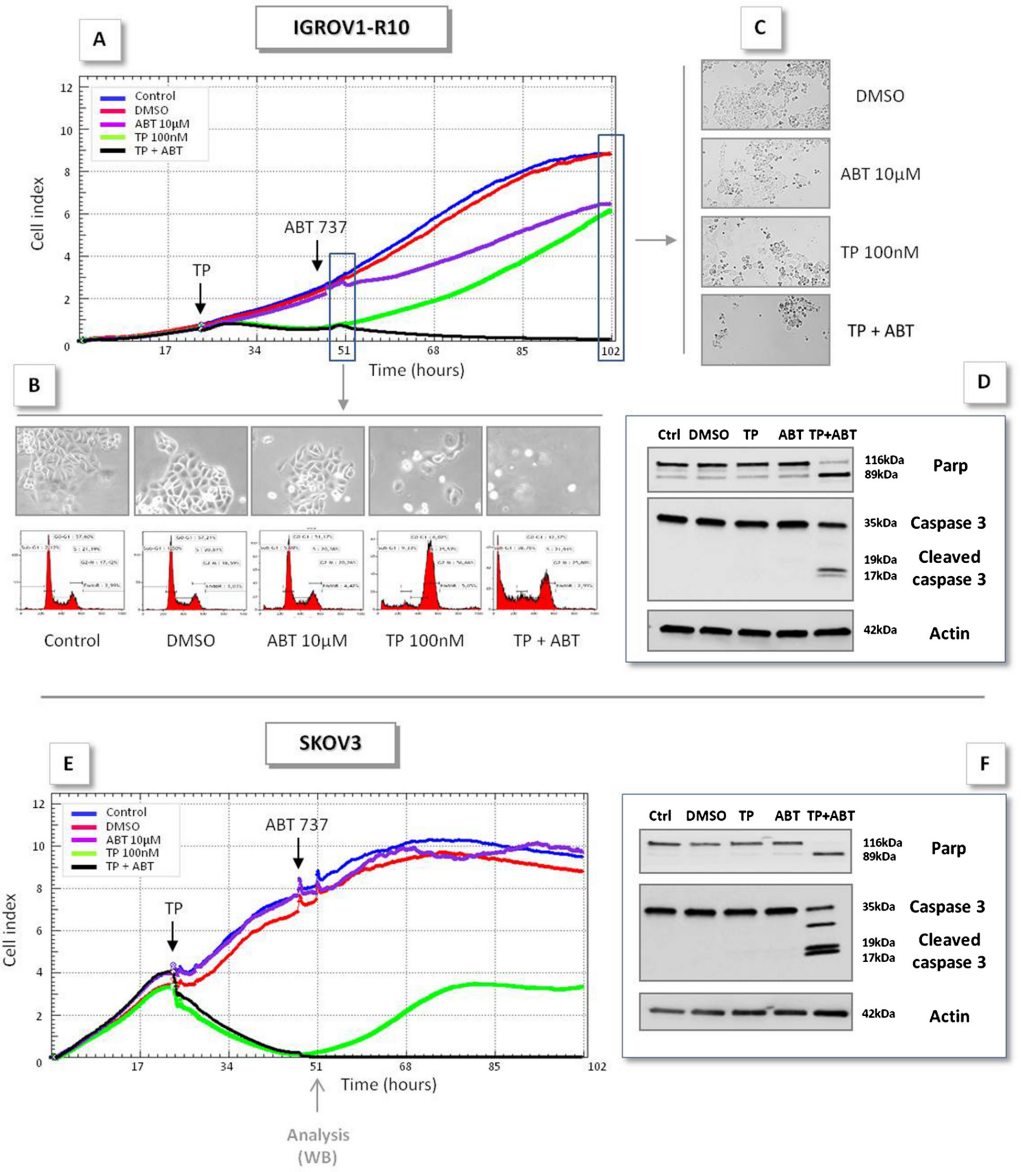
**MR22388 sensitizes ovarian cancer cells to ABT-737.** Real time analysis of cellular activity of the combination of MR22388 and ABT-737 was performed using the xCELLigence system. Cells were grown for 24 h, then treated with MR22388 (10, 100 and 1000 nM). 24 h after the beginning of the exposure, IGROV1-R10 (top panel) and SKOV3 (bottom panel) were treated by 10 μM ABT-737. [**A,E**] Cell index, recorded every 30 min. The results are the mean of three replicates. [**B**] 4 h after the ABT-737 exposure, IGROV1-R10 DNA content histograms and cellular morphology were assessed. [**C**] 72 h after ABT-737 exposure, IGROV1-R10 cellular morphology was assessed. PARP and Caspase 3 cleavages were determined by western blot in IGROV1-R10 [**D**] and SKOV3 cells [**F**].

After a 24 h exposure to MR22388 (when Mcl-1 expression is decreased), cells were treated by ABT-737. Whereas 100 nM MR22388 alone allowed the recovery of proliferative population, as previously evocated, its association with ABT-737 suppressed this recovery in both cell lines (Figure 3A and 3E), leading to the complete annihilation of the population. In contrast, ABT-737 had no (SKOV3) or little (IGROV1-R10) effect as a single agent.

This strong cytotoxic effect of the association is confirmed by morphological observations (Figure 3B and 3C). Moreover, the combination MR22388/ABT-737 led to a complete PARP and Caspase-3 cleavage, evocative of massive cell death (Figure 3D and F), whereas MR22388 or ABT-737 alone did not. Similar results were obtained on both cell lines using siRNA directed against Bcl-x_L_ instead of ABT-737 (data not shown).

## Discussion

The tripentone MR22388 has been identified as a potential anti-tumour agent and demonstrated strong *in vitro* anti-proliferative and cytotoxic activities on various cell types [10,15,16]. We observed similar effects in two chemoresistant ovarian cancer cell lines. MR22388 exposure induced strong cell cycle perturbations and triggered apoptosis in a concentration dependent manner.

MR22388 has been shown to be able to exert various effects such as anti-tubulin effects [15] and inhibition of GSK3, CDK1 [11] and showed an affinity for FLT3-ITD [10].

However, whatever are the MR22388 direct targets, we focused our attention on possible indirect effects of MR22388 on Bcl-2 family members since we previously demonstrated that anti-apoptotic proteins Bcl-x_L_ and Mcl-1 cooperate to protect ovarian carcinoma cells against apoptosis. Indeed, their concomitant inhibition leads to massive cell death, even in absence of chemotherapy [6,7]. Here we showed that Mcl-1 protein (but not mRNA) was drastically decreased in both cell lines after exposure to MR22388.

Mcl-1 is submitted to a rapid turnover, and the control of its expression could involve both transcriptional and post-translational mechanisms. Its interactions with other Bcl-2 family members can favour its stabilization or its degradation, depending of the partner [17]. Mcl-1 degradation can involve the proteasome pathway following JNK and GSK3 associated Mcl-1 phosphorylations [18], but can also be a consequence of caspases activity [19]. This last possibility has been excluded in our model since Mcl-1 was decreased even in condition in which apoptosis is not detected (24 h).

We also detected the appearance of the ser62-phosphorylated form of Bcl-x_L_. Phosphorylated forms have been previously described by others in response to various stresses [20,21]. Ser62-phosphorylated form has been associated with a loss of anti-apoptotic function [22] and to the appearance of a capacity of phospho-Bcl-x_L_ to translocate in the nucleus to lead to a G2-M cell cycle arrest. Moreover, these events as well as Bcl-x_L_ ser62-phosphorylation have been related to the activation of JNK, among other kinases [23].

Interestingly, it has also been shown that phosphorylation of Mcl-1 by JNK (itself activated by various apoptotic stresses) is required for subsequent docking of GSK3 on Mcl-1 allowing Mcl-1 phosphorylation, ubiquitinylation and degradation [24].

Altogether, our observations (G2-M blockade, Mcl-1 expression decrease and phosphorylation of Bcl-x_L_ on ser62) argue in favour of an activation of cell-death related signalling pathways such as JNK pathway by MR22388.

In any case, the capacity of MR22388 to inhibit Mcl-1 could be positively exploited in the context of Bcl-x_L_-targeting strategies. Here we showed that the association of MR22388 with ABT-737 was highly cytotoxic, in a manner correlated with the disappearance of Mcl-1.

Here we could hypothesize the existence of cooperation between tripentone that decrease Mcl-1 expression level and ABT-737 that inhibits Bcl-x_L_ and consequently releases the BH3-only protein Bim from Bcl-x_L._ Bim subsequently becomes able to inactivate residual Mcl-1 protein, and to activate Bax/Bak apoptotic proteins. Indeed, a shuttling of Bim sequestration between Mcl-1 and Bcl-x_L_, depending of their respective expression level has been recently described [25], Bcl-x_L_ being here presented as a determinant of the response to Mcl-1 inhibiting agents. In our model, MR22388 and ABT-737 should thus be considered as co-sensitizer agents.

In summary, although MR22388 activity is not yet fully understood, its interesting effect on the modulation of Bcl-2 family proteins could be positively exploited in the context of Bcl-x_L_-targeting strategies such as emerging BH3-mimetic molecules.

## Abbreviations

ABT-737: ABBOTT molecule 737; Bcl-2: B-cell lymphoma protein-2; Bcl-xL: B-cell lymphoma-extra large; BH3: BCL-2 homology domain 3; Bim: B-cell lymphoma 2 interacting mediator of cell death; CDK1: Cyclin-dependent kinase 1; DAPI: 4′,6′, Diamidino-2-phenylindole; FLT3-ITD: Fms-Like Tyrosine kinase 3 with an internal tandem duplication; GSK3: Glycogen synthase kinase 3; JNK: c-Jun N-terminal kinases; Mcl-1: Myeloid cell leukemia 1; PARP: Poly (ADP-ribosyl) polymerase; TP: Tripentone MR22388.

## Competing interests

The authors declare that they have no competing interests.

## Authors’ contributions

JT carried out and coordinated experiments and results analysis, and participated to the construction of the manuscript. AMF participated in study design and FG carried out the western blot assays. EB carried out xCELLigence studies. MHL performed RT-qPCR assays. EA carried out cell culture and assisted xCELLIgence studies. SR coordinated all studies concerning MR22388. PG and LP conceived the study, participated in its design, drafted the manuscript and coordinated its writing. All authors read and approved the final manuscript.
